# Electron evolution around a repulsive dopant in a quantum wire: coherence effects

**DOI:** 10.1039/c8nr06933f

**Published:** 2018-11-16

**Authors:** Mauro Ballicchia, Josef Weinbub, Mihail Nedjalkov

**Affiliations:** a Institute for Microelectronics , TU Wien , Austria . Email: m.ballicchia@gmail.com; b Department of Information Engineering , Università Politecnica delle Marche , Italy; c Christian Doppler Laboratory for High Performance TCAD , Institute for Microelectronics , TU Wien , Austria

## Abstract

We present an analysis of the quantum processes involved in the electron evolution around a repulsive dopant in a quantum wire. The quantum electron behavior has been studied by using a Wigner function approach. The Wigner phase space description allows the treatment of both classical and quantum evolution in the same framework, enabling ease of highlighting the effects of coherence. While the former is governed by a force, which is the first derivative of the dopant potential, the latter accounts for the entire potential, namely all derivatives in the corresponding Taylor expansion take part in the interaction. This gives rise to processes of tunneling and non-locality of the action of the potential. The complicated interplay of these quantum effects with the boundary conditions associated with the wire affects the physical observables like electron and current densities and in particular can give rise to an increase of the total current.

## Abstract 2

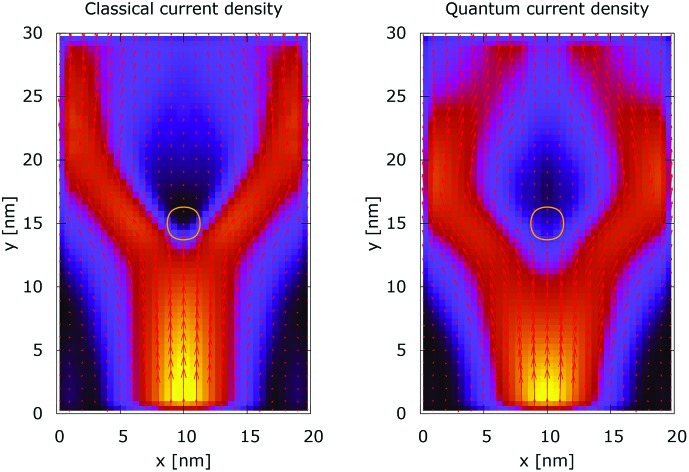
The interplay of coherence effects, like non-locality and tunneling, generates a quantum current density path around a repulsive dopant that is much more efficient than in the classical case.

## 1. Introduction

Recent developments in doping technologies enable new approaches to atomic-scale fabrication that allows few or single dopant atoms to be placed with nanometer precision in, *e.g.*, crystalline silicon or other materials.^1^–^3^ This makes dopants potential, promising building blocks for realizing future quantum devices. It is therefore evident that understanding the fundamental properties of individual dopants locally coupled to electronic transport in semiconductors is of particular importance and a key enabler to develop new devices based on quantum mechanical principles.^4^–^6^ The aim of this paper is thus to analyze the manifesting quantum effects in the evolution of electrons in the presence of a dopant inside a quantum wire.

### Modeling of classical and quantum processes

Investigating the differences between the quantum and the classical transport of charged particles in semiconductors is a fundamental challenge in modern physics and nanoelectronics. The continuous downscaling of device dimensions makes quantum effects even more evident, since in the nanometer and sub-nanometer regime electrons can no longer be considered point-like particles, and their finite-size and their wave-like nature should be accounted for. Thus using a quantum mechanical description becomes essential to correctly model the transport processes in modern nanostructures. Besides miniaturization problems, managing quantum effects becomes an opportunity for designing conceptually novel devices along with introducing a new engineering discipline based on the key quantum notions such as coherence, interference and entanglement. Recently the term *entangletronics* (short for entangled electronics) has been proposed based on the analysis of electron state control by lens-shaped potentials.^7^ Here, entanglement is not strictly related to the quantum entanglement of two or more correlated particles, where the quantum state of one of them cannot be described independently from the state of the other(s), but unifies a much broader class of problems, based on the processes of coherence. Indeed it has been recently demonstrated that coherence and entanglement are quantitatively equivalent,^8^ and a finite amount of coherence in a system can be converted into an equal amount of entanglement between that system and another initially incoherent system. So, entangletronics covers all mechanisms which maintain coherence. Based on this initial work on lens-shaped potentials and also within the scope of entangletronics, more recently, interference effects manifesting in a double-dopant potential structure have been investigated.^9^

In nanoelectronics we deal with the electron system in a *device*, which is correlated to electron states in systems called *contacts*, which account for the more or less coherent *communication* with the external environment. This communication is established *via* the *boundary conditions*, which, as will be further discussed, play a controlling role for the coherence of the electron transport. Internal processes of interaction with the lattice imperfections of the device crystal, called scattering events, as a rule cause decoherence. The latter is intimately close to the concept of reversibility: processes causing loss of coherence give rise to irreversible evolution.^10^–^13^

We consider the fundamental problem of a single electron evolving under the action of a scalar potential. From a formal point of view, classical and quantum transport descriptions of this problem are already very different. Nevertheless we will see that both descriptions can be approached by a common set of concepts and notions.

Classical statistical mechanics is governed by the Boltzmann equation that provides a statistical description of the system in the phase space based on a classical probability distribution. The Boltzmann distribution can be interpreted, both as the probability to find a point-like particle in a specific state (a specific position and a specific momentum or velocity) or equivalently as the mean number of many non-interacting particles in an infinitesimal interval around a specific state. This property reflects the spatially local nature of the classical transport that involves only the electric force, *i.e.*, the first derivative of the electric potential and the processes of scattering.

In this framework the interplay of different phenomena could be simplified by analyzing each of them separately and finding the corresponding probability. The overall interaction of these phenomena is determined by a cumulative sum of the relative probabilities. An example of this approach is the use of the Matthiessen rule for the derivation of classical electron mobility.^14^,^15^

Quantum mechanics is based on the phases and amplitudes of the wavefunctions, and their interplay gives rise to interference, non-local effects, and tunneling phenomena that cannot be described in the phase space in terms of probabilities. A small change of the physical conditions can cause a dramatic change in the interference pattern, which makes the study of such phenomena so difficult. A quantum process cannot be simplified into elementary processes with associated probabilities, which can be studied separately, and then added to provide a cumulative description of the system evolution. It should thus be considered as a whole when conducting quantum transport analysis. Furthermore, the description is potential-based: quantum evolution is not governed only by the first derivative as in the classic case, but all derivatives in the Taylor expansion of the potential take part. This means that if the electric potential is a linear or a quadratic function of the position within the wavefunction extension, the transport is classical, along Newtonian trajectories. Abrupt variations of the electric potential that exceed the quadratic dependence, give rise to non-local effects, tunneling effects, and may also give rise to interference phenomena. In particular the Coulomb potential of an ionized impurity (dopant) features a *beyond quadratic* variation in space and thus different quantum and classical evolutions.

As previously stated, two close settings of the physical parameters can cause strongly different behavior in the quantum evolution. This makes numerical modeling a fundamental tool for studying quantum phenomena. In particular modeling allows one to consider specific conditions that are difficult to implement by experimental approaches, or to selectively consider specific properties of the simulation setup, avoiding the change of the other parameters. Emblematic examples are the options to suppress the effects of scattering or to take into account different kinds of injecting and reflecting boundary conditions. These considerations impose simulations as a research approach relevant for analysis of the electron evolution around a Coulomb impurity in a quantum wire. Moreover, to identify quantum effects, it is convenient to utilize the classical behavior as a reference frame. This motivates the choice of the Wigner formulation of quantum mechanics,^16^ which incorporates many classical aspects in contrast to other formalisms, where states have real and imaginary components and physical observables are described by operators that act on them.

Indeed, the Wigner theory bears the canonical concepts of phase space and a (quasi-)distribution interpretation: the operators representing the physical observables are replaced by the corresponding real functions of the phase space variables. These functions allow the evaluation of the mean value of the physical observables in the same fashion as in classical statistical mechanics; despite that the Wigner function can assume negative values. The formalism gradually recovers the classical evolution rules already in the ballistic case: for slowly varying potentials the Wigner equation reduces to the ballistic Boltzmann equation, which demonstrates the full correspondence between the two sets of evolution concepts. Importantly, the Wigner evolution continues to be fully quantum so that the difference between the two pictures is introduced by the initial condition, which needs to reflect the uncertainty relations.

The Wigner function has found broad application in science and engineering,^17^ in particular in recent years.^18^

In what follows we use a stochastic interpretation of the Wigner formalism^19^ which brings the two pictures even closer by further extending the particle nature of the classical mechanics for the quantum case. As discussed, electron–electron interactions are ignored at this fundamental level of description, but can affect the electron evolution *via* screening of the electric potential. The effects caused by the processes of decoherence are also beyond the scope of this work. Indeed, before such analysis we need to know what coherent phenomena are destroyed by these processes, which is the core focus of this work.

A specific reformulation of the Wigner transport model is provided by the signed particle approach. The concepts of signed particles have been first introduced for stationary transport^20^ and further extended for transient problems.^21^,^22^ The Wigner function is modeled by stochastic numerical particles, which evolve in the phase space, bearing most of the properties of the classical particle model, like Newtonian trajectories and ensemble averaging. However, novel concepts, such as particle sign, and evolution rules, such as generation and annihilation, are introduced to account for the quantum information in the system.

This framework adopts many notions of the classical counterparts and allows ease of switching between classical and quantum rules. Moreover it allows the simulation of the quantum particle evolution using different boundary conditions^23^ to study the interplay between evolution and boundary processes.^24^

### Physical problem and approaches

We start with formulating the main contributions of this work. The aim is to analyze the quantum effects in the evolution of a single electron in the presence of a repulsive dopant inside a quantum wire. These effects are relevant in the limit where the discrete nature of the electron charge cannot be ignored: at this limit the single electron evolution determines the involved physical effects.^25^ The physical system consists of a portion of the quantum wire with a repulsive dopant placed in the center. Electrons are injected from the bottom of the quantum wire and flow to the top of the quantum wire, interacting with the dopant. Two types of boundary conditions are considered (a) all absorbing lateral boundaries; (b) specularly reflecting lateral boundaries.

The numerical simulation framework, based on Wigner signed particles and implemented in viennawd,†
†http://www.iue.tuwien.ac.at/software/viennawd/ allows switching between classical and quantum evolution rules and between different boundary conditions. In the case of lateral absorbing boundary conditions, the interaction with the dopant governs the electron evolution.

The boundary conditions come into play by switching from absorbing to reflecting boundaries, which is the main feature of a quantum wire. However a number of boundary models can be specified to reflect the concrete physical conditions which introduce specular reflection or quantum penetration or surface roughness or random (stochastic) reflection. We chose the first option, corresponding to an infinite, ideal potential defining the wire, which resembles the classical boundary model used in device Monte Carlo simulations.^26^ In this way we avoid the effects due to quantum penetration in the walls,^27^–^36^ which can't be accounted for in the reference classical simulations.

Since we are interested in highlighting the quantum effects, phonon scattering has been suppressed, both in classical and quantum simulations, even if the simulation framework is able to model this phenomenon.^22^ In this way we avoid processes of decoherence^37^ and achieve maximum resolution in comparing classical and quantum simulation results.

The simulation setup resembles the double-slit experiment, where single electrons are shot consecutively towards a screen, until an interference pattern is obtained. The probabilistic interpretation is of equivalent independent (uncorrelated) experiments aiming at accumulating sufficient statistics. The pattern is independent of the time between the consecutive injections, provided that the consecutive electron evolution events are uncorrelated. Accordingly, we inject inside the simulation domain Wigner states, corresponding to equivalent minimum uncertainty wavepackets. Such injection gives rise to a time-averaging of the physical quantities of the individual states such as the density and current given by eqn (5) and (6). The evolution of the consecutively injected states is uncorrelated: a self-consistent evolution accounting for electron–electron interaction *via*, *e.g.*, the Poisson equation corresponds to an entirely different physical problem. The simulation continues until a stationary picture is obtained, which indicates accumulation of sufficient statistics, similar to the double-slit experiment.

In the classical case we use the same injection conditions, but in this case these *wavepackets* follow a Gaussian charge distribution.

In the next section, section 2, we introduce the Wigner formalism. The simulation setup is presented in section 3. Finally, the analysis identifies phenomena related to tunneling and quantum non-locality (section 4). Their interplay with the boundary reflection in particular leads to an enhancement of the current through the wire. The results give insight into the not well understood quantum transport phenomena in quantum wires.

## 2. The Wigner formalism

### Quantum mechanics in phase space

We present the basic peculiarities of the chosen phase space formulation of quantum mechanics. Historically the Wigner function^16^ has been introduced *via* the density matrix, and thus all further theoretical notions are derived on top of the operator mechanics. It took more than three decades to prove that the Wigner theory allows for an independent formulation and to show that it is fully equivalent to the operator mechanics, by deriving the latter from the phase space quantum notions.^38^–^44^ As this way relies on mathematical abstractions like the Moyal bracket and the star product we prefer the more intuitive historical way to comment on the basic notions of the phase space theory. In the Wigner picture both observables and states are functions of the variables of the phase space: the position and the momentum. The main goal is to establish a map between the wave mechanics operators *Â*(*r[combining circumflex]*, *p[combining circumflex]*) and the real functions *A*(*r*, *p*), which is hindered by the fact that position and momentum operators do not change.

Algebraically equivalent functions of these variables give rise to different operators, that is, the set of operators is larger than the set of functions. It appears that it is sufficient to select a subset of the full set of wave operators to develop a physically meaningful theory. The Weyl map1
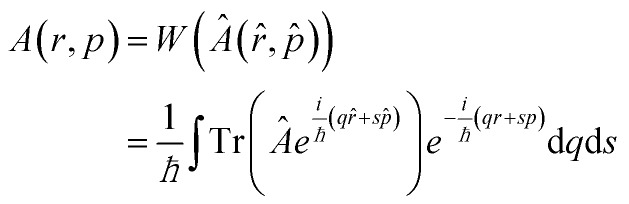
establishes a correspondence between phase space functions and operators, expressed by position and momentum operators, which appear in as a fully symmetric order. The Wigner function is the Weyl map of the density matrix *ρ*(*r*, *r*′, *t*) = ) = 〈*r|φ*_t_〉〈〉〈*φ*_t_|*r*′′〉 = = *φ*(*r*, *t*)φ*(*r*′, *t*) and can be expressed as:2
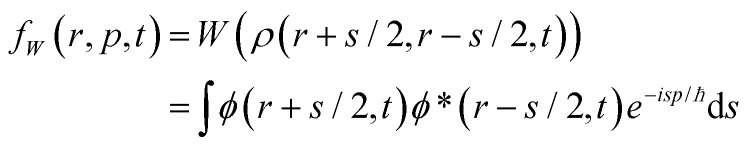



In this way not all phase space functions are admissible states. The latter need to reflect the uncertainty relations *via* the density matrix. The Wigner function *f*_W_ is a real function, which can have negative values, but retains the basic properties of the classical statistical distribution. Physical averages can be obtained from it in the same way as in classical statistics. In particular, the most important property of the Wigner function is that the mean value of a physical quantity is given by3
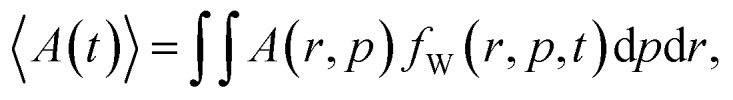
allowing it to be called a *quasi-distribution*. Indeed, if the Weyl map is applied to the principal expression of the expectation value . Indeed, if the Weyl map is applied to the principal expression of the expectation value 〈*A*〉 = Tr( = Tr(*Âρ̂*), the trace operation turns into an integration over the phase space, the density operator becomes the Wigner function, and *Â* maps into the same classical function, which represents a generic physical quantity as energy or velocity. It is important to note that the Weyl map is not unique: other correspondence rules can be formulated, where for example positions precede momenta (standard order), or *vice versa* (anti-standard ordering). The choice of alternative maps leads to different quasi-distributions. It should be noted that once postulated, the correspondence rule must be consistently applied to all notions of the operator mechanics and in particular to the trace, which means that both integrands in (3) are map-dependent.

Applying the Weyl transform to the Von Neumann equation provides the equation of motion for the quasi-probability distribution – the Wigner equation.4
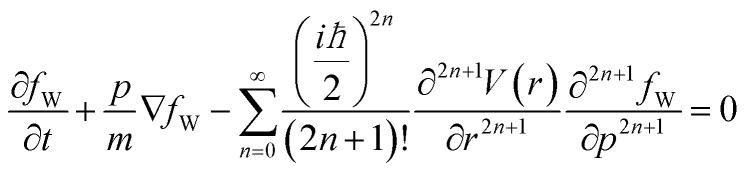



The evolution depends on all odd derivatives of the potential. To switch from the quantum to the classical rules it is sufficient to consider only the first derivative of the potential: in this way the Wigner equation reduces to the classical Boltzmann transport equation, where the particle evolution is governed by the local force. The here used simulation framework is able to model this phenomenon.

Without phonon scattering and other sources of dissipation, the total energy of the interaction with the dopant potential is conserved in both classical and in quantum cases. Thus it is sufficient to consider the first two moments of *f*_W_, namely the electron density *n*(*r*, *t*) and the current density 
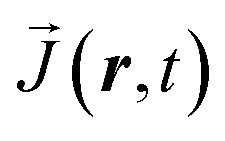
 :5
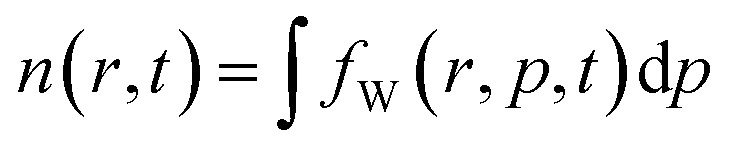

6
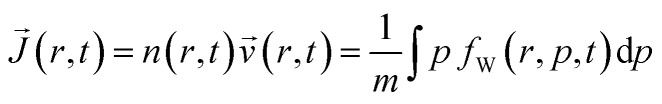



The same relations hold also in the classical case, where *f*_W_ is replaced by the corresponding distribution function.^45^

### Wigner signed particles

The concept of Wigner signed particles comprises a set of attributes, which can be combined into a variety of algorithms suitable for modeling particular transport tasks. In the following, we summarize these concepts, which are needed for our analysis. Signed particle attributes are based on the numerical Monte Carlo theory for solving integral equations,^46^ applied to different integral forms of the transport task^47^ – transient^21^ or stationary,^20^ in the presence of initial or/and boundary conditions.

The Wigner equation can be formally written as a second kind of a Fredholm integral equation:
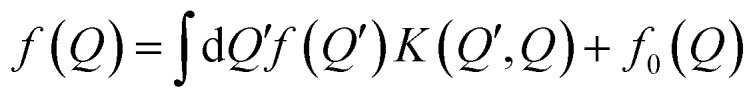
having a solution *f*, which can be presented as a series of consecutive iterations of the kernel *K* on the free term *f*_0_: 
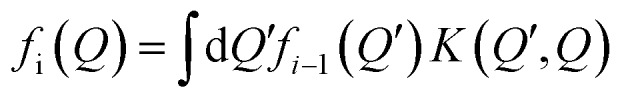
. The last integral can be presented as an expectation value of a random quantity with a probability distribution *P*(*Q*, *Q*′):7
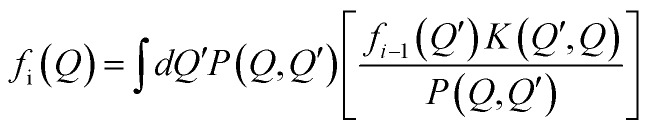



Here the function *P* is a probability distribution with respect to the variable *Q*′ for any fixed value of *Q*. According to numerical rules,^48^*P* is used to sample points *Q*′, where the values of the random variable given by the term in square brackets in (7) (called weights) are calculated. Their mean value approximates the expectation value *f*_i_, as asserted by the central limit theorem.^49^ This procedure can be generalized for calculating the sum *f*: due to the consecutive appearance of the kernel, the probability function *P* can be used repeatedly in the following scheme: the initial point where *f* is evaluated is fixed, and then *P*(*Q*, *Q*_1_) generates the next point *Q*_1_, which is used to generate *P*(*Q*_1_, *Q*_2_) *etc*. The product of the consecutive weights is called total weight, which is the random variable for the sum, and the consecutive points *Q*_i_ are called numerical trajectory. Accordingly, the trajectory is associated with a numerical particle.

In this spirit, the mean value of a generic physical quantity *A*, can be presented as a series 
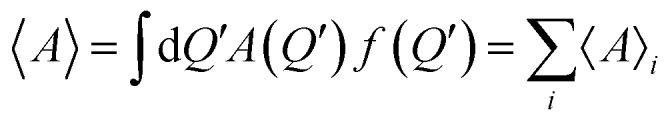
 where the terms where the terms 〈*A*〉_*i*_ are obtained by the consecutive iterations of the kernel of the integral form of the Wigner equation on the free term, given by the initial or the boundary conditions.

To continue, we need to specify the physical conditions. We focus on problems for evolving an initial condition described by the time-dependent Wigner equation. The point *Q* in this case is comprised by the phase space and time coordinates. The concepts of signed particles are introduced similarly to the stationary counterpart,^50^ but the evolution is performed at consecutive time steps.

The kernel *K* can be simplified into a sum of conditional probabilities, which is then used to construct the function *P* = {*P*_w_^+^ + *P*_w_^–^ + *P*_δ_} as a sum of three probabilities. The first two correspond to the generation of two particles in two novel phase space points. Since the kernel has the same structure as *P*, with the difference that the sign in front of *P*_w_^–^ is minus, a weight factor of –1 appears in (7) when *P*_w_^–^ is applied. This weight can be associated with the generated particle, giving rise to a picture of positive and negative particles evolving in the phase space.

The kernel of the Wigner equation provides the quantum information *via* the generation process. The particle sign carries the quantum information, which is taken into account *via* the mean value of the physical averages. This allows for another key concept – particle annihilation: particles with opposite signs which meet in phase space *at the same time* annihilate each other. The annihilation property is crucial for avoiding the divergence of the number of generated particles.

The here introduced signed particle approach enables considerable physical insights into various quantum mechanical processes and will be used for the subsequent analysis.

## 3. Experimental setup

**Fig. 1:**
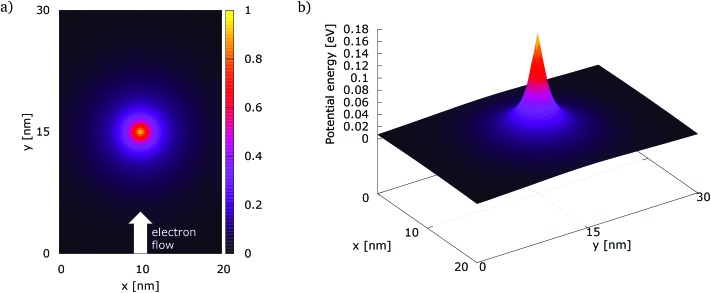
The simulation setup with: (a) the normalized potential energy map of the dopant, (b) the potential energy of a dopant with a peak energy of 0.175 eV.

The simulation setup consists of a quantum wire with the extent of 20 × 30 nm^2^. A repulsive dopant is placed in the center of the wire at *r*_d_ = (*x*_d_, *y*_d_) = (10 nm, 15 nm) and is modeled by its Coulomb potential. Fig. 1(a) shows the simulation setup with a map of the potential energy of the dopant normalized with respect to its peak value. Fig. 1(b) shows the potential energy of a dopant with a peak energy of 0.175 eV.

Indeed the repulsive dopant is modeled by a screened Coulomb potential8
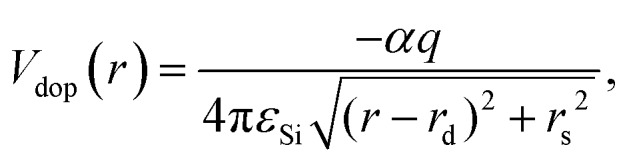
where *r*_s_, the screening radius, removes the singularity of the Coulomb potential and fixes the peak value of the corresponding potential energy in *r* = *r*_d_. The aim of this parameter is to model the screening of the valence electrons against the dopant nucleus and fixes the radius at which the corresponding electric field assumes the maximum strength. The parameter *α* determines minimum of the Coulomb potential and the peak of the corresponding potential energy, that should correspond to the ionization energy.

In order to simulate the electron evolution around the dopant, electrons are injected at the bottom center of the simulation domain *r*_0_ = (*x*_0_, *y*_0_) = (10 nm, 0 nm), as shown in Fig. 1(a).

To investigate coherent transport in the quantum case, the initially injected states are identical minimum uncertainty wave packets, which are described by the following Wigner distribution:

9 *f*_W_(*r*, *k*) = *N* exp{–|*r* – *r*_0_|^2^/(2*σ*^2^)}exp{|*k* – *k*_0_|^2^2*σ*^2^}

which is an admissible Wigner pure state^51^ corresponding to a minimum uncertainty wave-packet. The width of the initial states in the position space is set to *σ*_*x*_ = *σ*_*y*_ = *σ* = 3 nm.

The minimum uncertainty wave-packet is characterized by a Gaussian distribution of the momentum with constant variance that is determined by the variance of the corresponding components in the position space: *σ*_*k*_*x*__ = 1/(2*σ*_*x*_) and *σ*_*k*_*y*__ = 1/(2*σ*_*y*_). Injecting initial states with constant variance in the momentum distribution, established according to the uncertainty principle, is of fundamental importance for studying coherence. Indeed setting the variance of momentum distribution according to a Fermi–Dirac or a Maxwell–Boltzmann distribution corresponds to the injection of mixed states and the introduction of decoherence in ballistic devices.^24^

In accordance with the single-electron picture, if we consider each wavepacket as an electron, the charge densities involved in the simulations are small and self-consistence effects are negligible. Indeed, injecting wavepackets with *σ* = 3 nm, yields an electron density of about 2–5 × 10^–20^ C nm^–2^ that generates potential variations of about 5 × 10^–10^ V nm^–2^, corresponding to variations of no more than 3 × 10^–7^ V in the whole simulation domain.

In the signed particle formulation of the Wigner formalism, each of these initial states is realized by generating a certain number of signed numerical particles, in this specific case 5 × 10^5^. The individual position and momentum is generated randomly according to the corresponding Gaussian distribution of (9). So, if we evaluate the mean and the standard deviation of the numerical particle positions, we obtain *r*_0_ and *σ*, respectively. In the same fashion if we evaluate the mean value and the standard deviation of the numerical particle momenta, we obtain *k*_0_ and 1/(2*σ*).

In the signed particle formulation the momentum space is discretized with a square mesh Δ*k* that is set on the base of the coherence length *L*_c_ by the relation Δ*k* = *π*/*L*_c_.

In all the simulations described in the following, a minimum uncertainty initial electron state is injected every 1 fs in the direction +*y*, with an initial kinetic energy of 0.141 eV, that corresponds to the set *k*_0_ = (*k*_0,*x*_, *k*_0,*y*_) with *k*_0,*x*_ = 0 nm^–1^ and *k*_0,*y*_ = 12Δ*k*, considering the electron effective mass m* = 0.19*m*_electron_ at a temperature of *T* = 300 K, and the coherence length *L*_c_ = 45 nm. The coherence length has been set to a value greater than the largest extension of the simulation domain, that is 30 nm, in order to have coherent transport inside the whole simulation domain, and corresponds to a minimal energy resolution of Δ*E* = *ħ*^2^Δ*k*^2^/(2m*) = 0.977 meV.

In the classical simulations the minimum uncertainty Wigner distribution, being positive, can be interpreted as a classical electron distribution that evolves according to the Boltzmann equation. So, in this way we can safely ensure equivalent injecting conditions for both, the classical and the quantum experiments, making the experimental setup identical in the two cases except eventually for the normalization condition.

Therefore, in this framework, the classical and quantum evolution differs only in the treatment of the dopant potential. As stated above, in the classical case only the first derivative of the potential is considered, which corresponds to give rise to a force and an acceleration along Newtonian trajectories, while in the quantum case higher order derivatives are taken into account *via* the Wigner potential, *cf*. the right-hand side of (4). The latter enables describing quantum effects.

## 4. Quantum effects

We first focus on the process of electron–potential interaction by switching off the reflecting boundaries.

### All absorbing boundaries

**Fig. 2:**
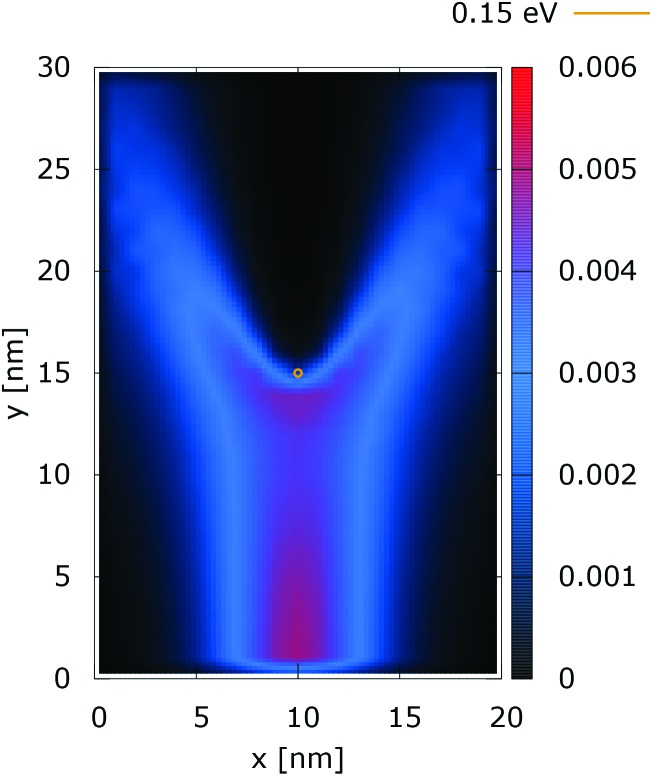
Classical electron density ([a.u.]). The dopant potential is 0.175 eV.

**Fig. 3:**
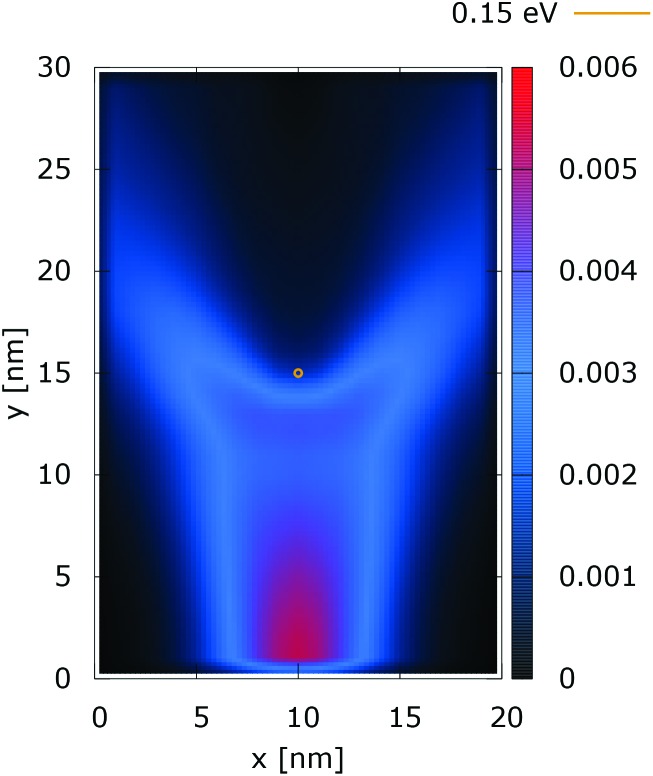
Quantum electron density ([a.u.]). The dopant potential is 0.175 eV.

Fig. 2 and 3 show the electron density for all absorbing boundary conditions in the case of a potential peak value of 0.175 eV. The electron evolution has been simulated starting with up to 400 fs evolution time. The evolution process reaches a steady state after 100 fs. The maps are obtained by averaging the simulation results from 100 fs to 400 fs. The orange circle in the middle of the figures shows the potential isoline at 0.15 eV, which clearly shows the position of the peak. The distribution in Fig. 2 can be understood in terms of the classical evolution. The consecutively injected particles from the bottom are distributed as Gaussian packets centered at *x* = 10 nm. Accordingly, above this point is a high density zone, marked in red. With the increase of the distance from the injecting boundary, particles spread to the left and right and thus the high density zone shrinks. The shape of the red region, which resembles a triangle, depends on the frequency of the injection of the consecutive packets and on the initial particle velocities (energies) in *x* and *y* directions. Particles with high *x* velocities spread further away from the *x* = 10 nm line and may leave the domain through the absorbing boundaries.

The rest of the particles approach the central region, where they begin to *feel* the repulsion of the Coulomb force. Their trajectories are modified so that the electron density forms a specific shape around the dopant. The red region in front of the center outlines a region of accumulation. In our experiment the initial kinetic energies have a mean value of 0.141 eV so that the majority of the particles can only *touch* the potential isoline. The black regions after the center indicate the forbidden zone for particles with kinetic energies less than the peak potential. Nevertheless, as we will show in what follows, this region is not entirely empty, as high energy particles from the tail of the Gaussian distribution can penetrate there. We note the sharp contrast between this V-shaped region with the two channels with blue color through which the deviated particles by the central force leave the region of interaction. With no force, particles recover their uniform motion, which gives rise to the linear shape of these channels.

The quantum evolution presented in Fig. 3 offers several peculiarities which are associated with the non-locality of the interaction and the effects of tunneling. Noticeably, the potential is felt immediately after the injection, as demonstrated by the modified density in the bottom region, far away from the center. Furthermore, there is a well visible zone of density depletion in the vicinity of the dopant. The processes of tunneling cause electrons to penetrate into the depleted region, which is well demonstrated by the smearing of the blue/black borders after the center. Both tunneling and non-locality give rise to the nonlinear shape of the two channels in the upper part of the figure.

**Fig. 4:**
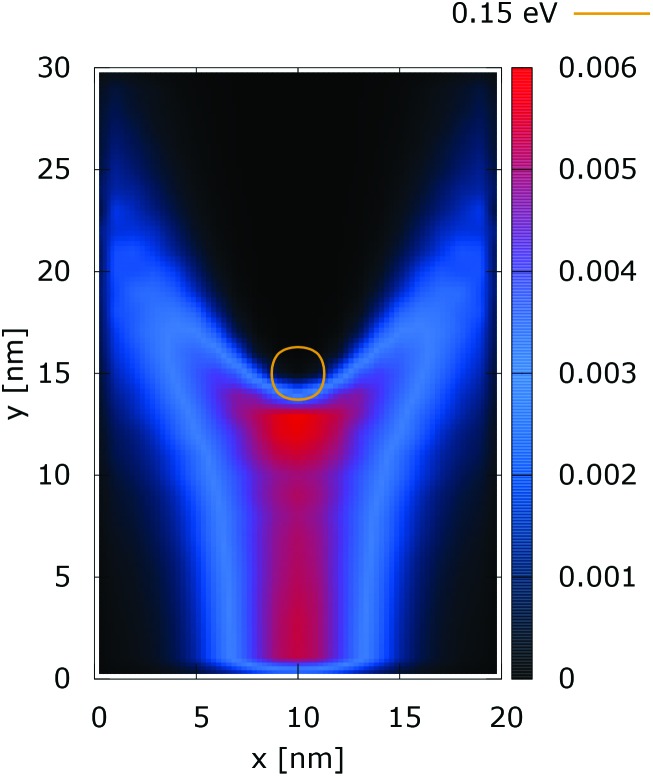
Classical electron density ([a.u.]). The dopant potential is 0.35 eV.

**Fig. 5:**
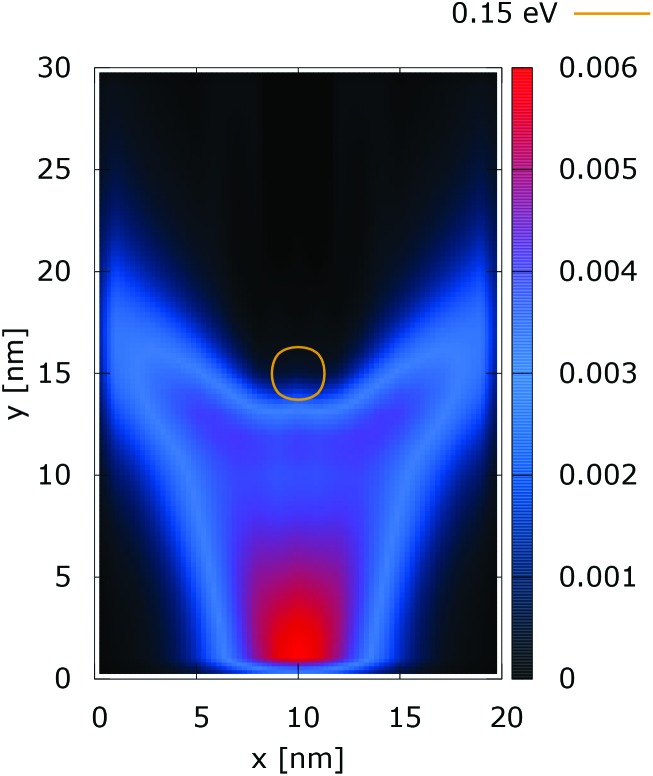
Quantum electron density ([a.u.]). The dopant potential is 0.35 eV.

These considerations are supported by further experiments, where the value of the dopant potential is doubled, while all other conditions remain the same. Fig. 4 and 5 show the classical and quantum electron densities for a potential peak of 0.35 eV. In particular, in the former case the density touches the 0.15 eV isoline again, while in the quantum counterpart the density stays away from the isoline. Higher potential values in the vicinity of the dopant give rise to a suppression of the tunneling, which allows the demonstration of the non-local action of the Coulomb potential. Also the modification of the density near the injection boundary is well pronounced.

**Fig. 6:**
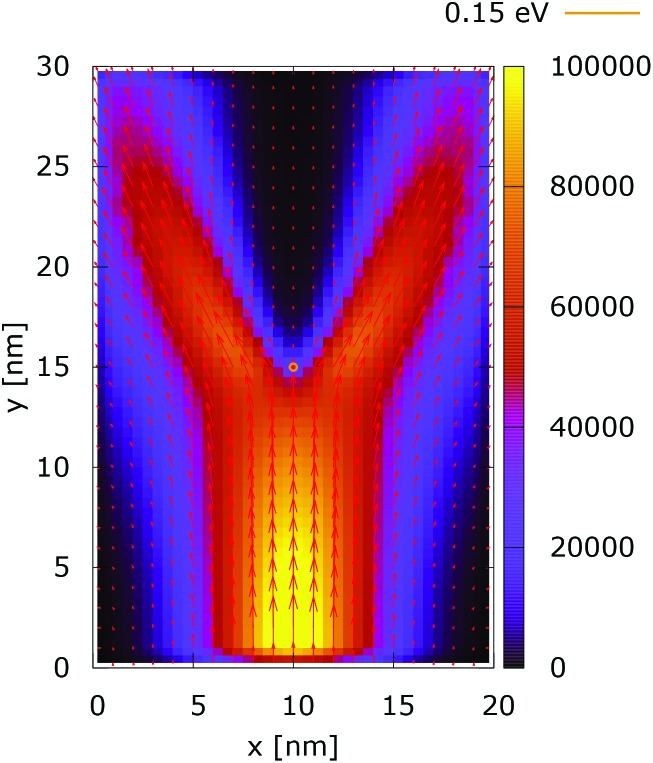
Classical current density ([a.u.]). The dopant potential is 0.175 eV.

**Fig. 7:**
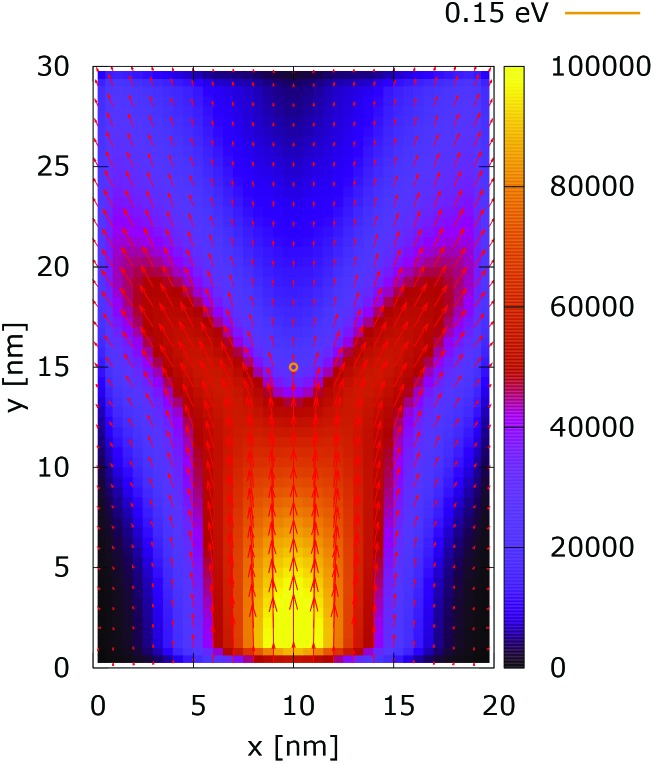
Quantum current density ([a.u.]). The dopant potential is 0.175 eV.

The processes of tunneling are better demonstrated in the current density plots, Fig. 6 and 7. The arrows show the direction of the local current, while their length is proportional to the corresponding magnitude. In the absence of current the arrows degenerate to dots, represented in the classically forbidden, black colored region in the upper central part of Fig. 6. In contrast, in the quantum case the presence of current is well demonstrated by both arrows and non-zero densities.

**Fig. 8:**
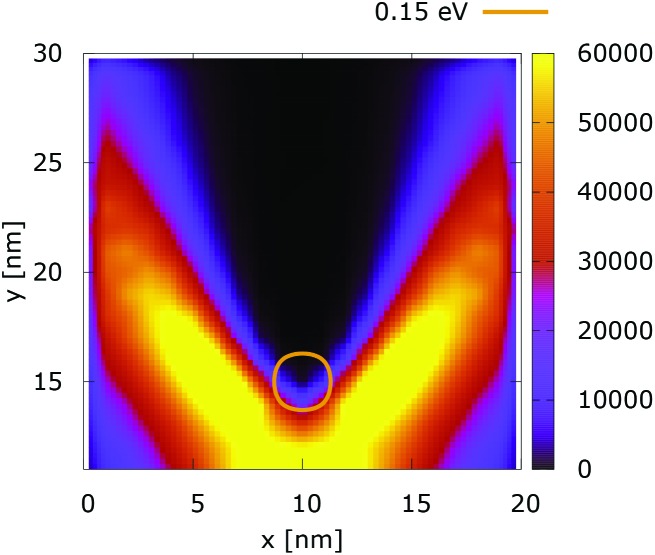
Classical current density [a.u.] in the case of 0.35 eV potential.

**Fig. 9:**
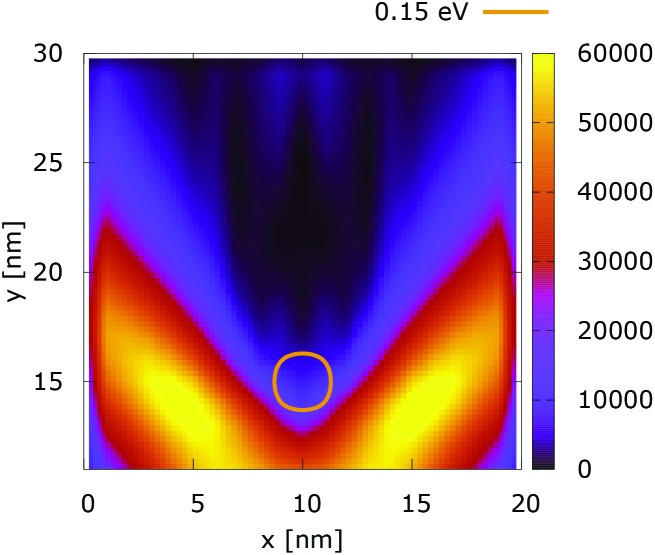
Quantum current density [a.u.] in the case of 0.35 eV potential.

Something very interesting is observed in the case of 0.35 eV, *cf*. Fig. 8 and 9. The top regions are enlarged and arrows are removed to demonstrate better the interplay between tunneling and non-local repulsion. The electron density in the area behind the center is not negligible due to the processes of tunneling. This density gives rise to local currents which increase further away from the dopant due to the repulsive character of the potential.

**Fig. 10:**
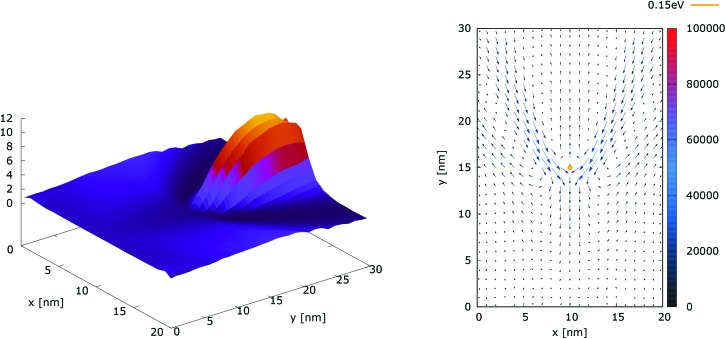
The quantum/classical current ratio (left) and the quantum-classical difference (right) in current densities for 0.175 eV.

Fig. 10 shows the ratio and the difference between quantum and classical current densities. Already the existence of the ratio prompts that the classical density in the V-shaped region is non-zero. Quantum current becomes one order of magnitude higher in the central part. At the borders of the region, however, the classical current density is higher. This is well demonstrated by the rotation of the current-indicating direction arrows towards the bottom in the plot with the current difference. Here, it is interesting to observe that the quantum non-local action is spread almost to the half of the distance to the injecting boundary. Indeed the bottom-pointing arrows around the central axis are spread down to the *y* = 7 nm line. The current due to tunneling clearly dominates in the V-shaped region, as the magnitude and orientation of the arrows prompt. In contrast, the classical current dominates at the border of this region, as suggested by the bottom-pointed direction of the arrows. These two opposite flows compensate in their net contribution to the total current, which is in contrast to the usual expectation that tunneling will effectively increase the current value.

### Quantum wire

Quantum wire boundary conditions are imposed by adding reflecting boundaries on the lateral sides (*x* = 0 nm and *x* = 20 nm). Boundary reflections interplay with the processes of tunneling and effects of non-locality in a rather complicated way.

**Fig. 11:**
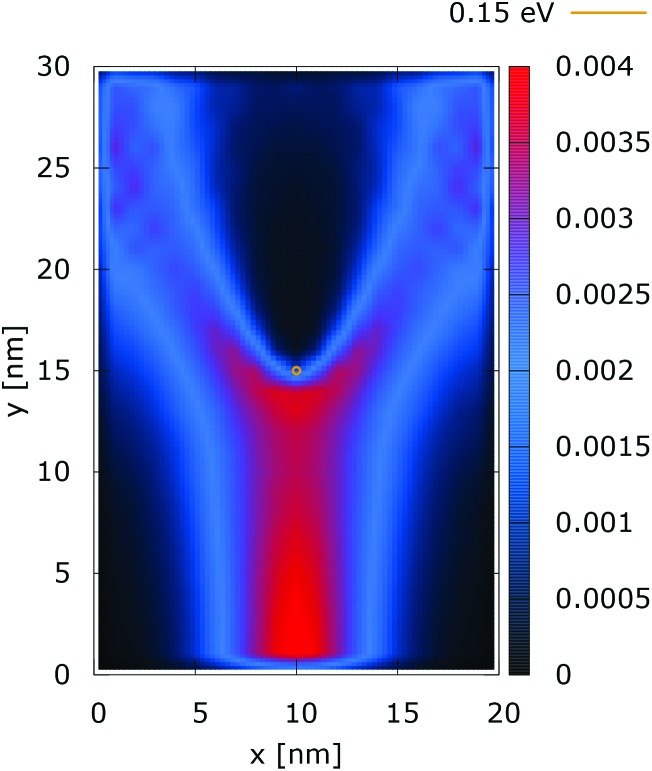
Classical electron density [a.u.] with lateral reflecting boundaries. The dopant potential is 0.175 eV.

**Fig. 12:**
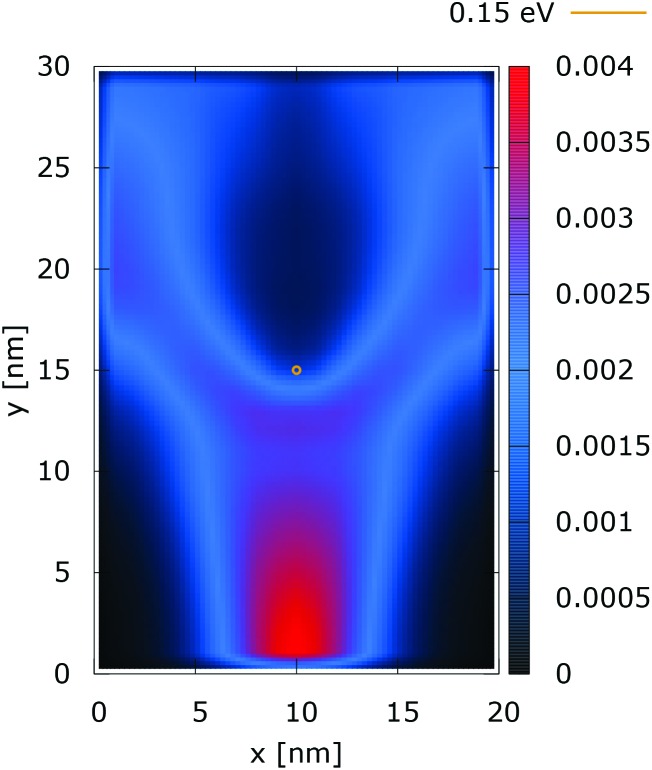
Quantum electron density [a.u.] with lateral reflecting boundaries. The dopant potential is 0.175 eV.

**Fig. 13:**
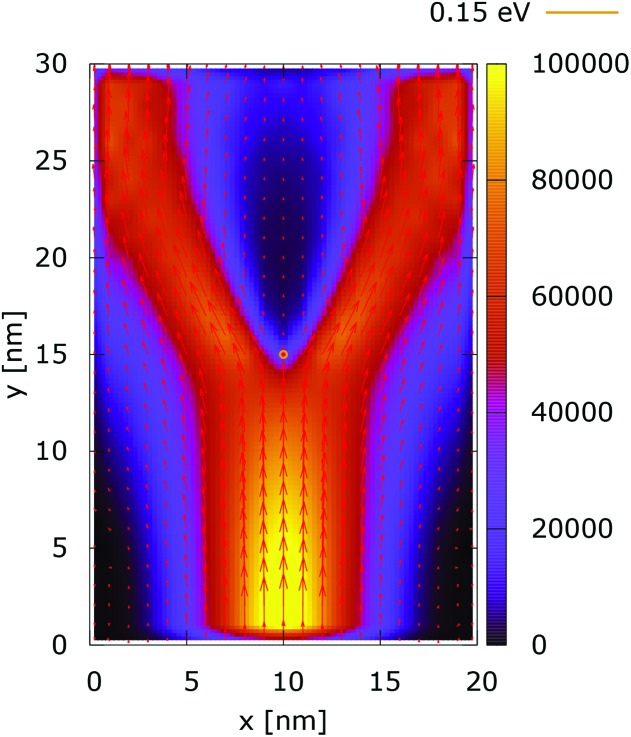
Classical current density [a.u.] with lateral reflecting boundaries. The dopant potential is 0.175 eV.

**Fig. 14:**
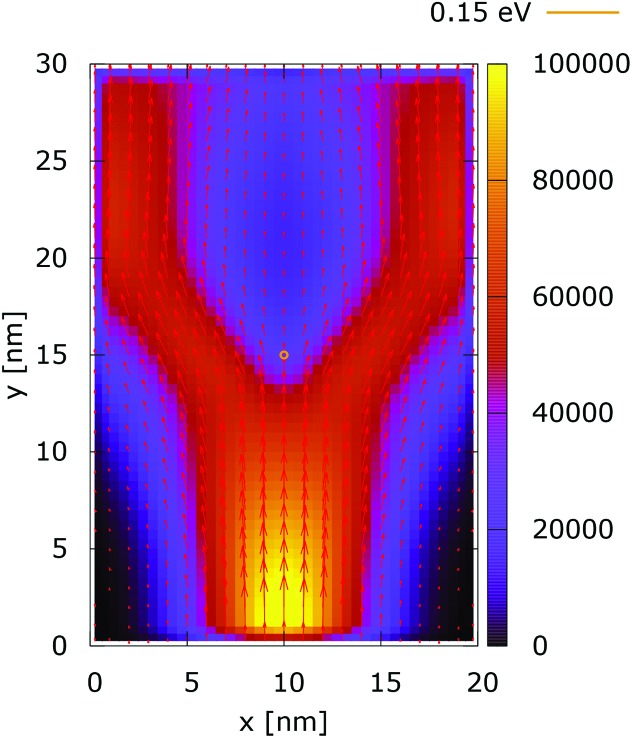
Quantum current density [a.u.] with lateral reflecting boundaries. The dopant potential is 0.175 eV.

Fig. 11 and 12 show the electron density with lateral reflecting boundaries for the 0.175 eV repulsive dopant. We first observe that the two channels surrounding the V-shaped region are not straight as their open boundary counterparts. This effect is attributed to the processes of reflection which occur around the *y* = 20 nm line. Another effect is due to the particles which scatter from the boundaries below the central part where the peak of the dopant is located. The border of the V-shaped region is not so sharp as in the open boundary case, since particles penetrate near the center, in the region between *y* = 15 nm and *y* = 20 nm. The effect of the processes of reflection is much stronger in the quantum case. In particular these processes begin further below the center due to the non-local action of the potential, which increases the flux towards the boundaries. The density in the V-shaped region is now much higher than in the classical case. This effect is better demonstrated in Fig. 13 and 14. In both cases the currents’ evolution paths are more closed as compared to the open boundary case. There is a significant increase of the current density right after the dopant in the quantum case. This is attributed to the joint effect of tunneling and repulsion, since the dopant potential repulses the penetrating electrons towards the boundaries earlier so that they reappear in the zone after the center sooner than in the classical case and thus are further accelerated by the repulsive (and more distant in the quantum case) action of the potential.

**Fig. 15:**
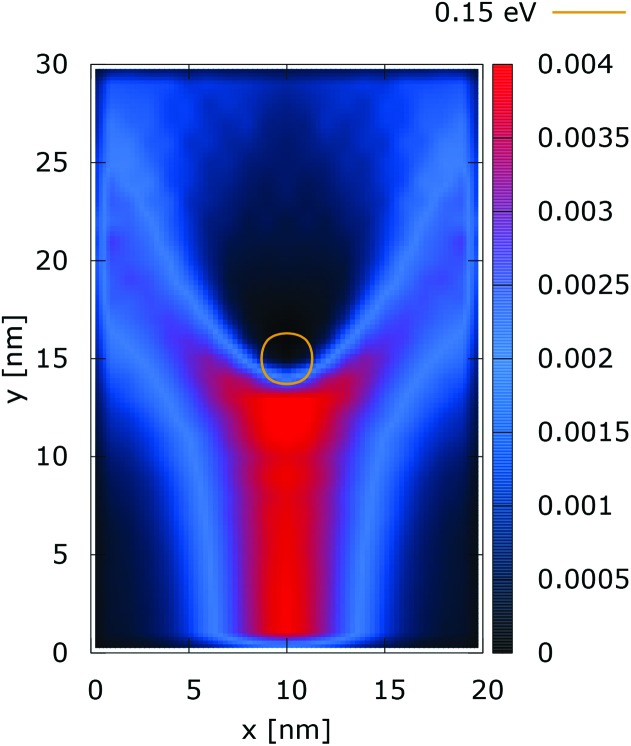
Classical electron density [a.u.] with lateral reflecting boundaries. The dopant potential is 0.350 eV.

**Fig. 16:**
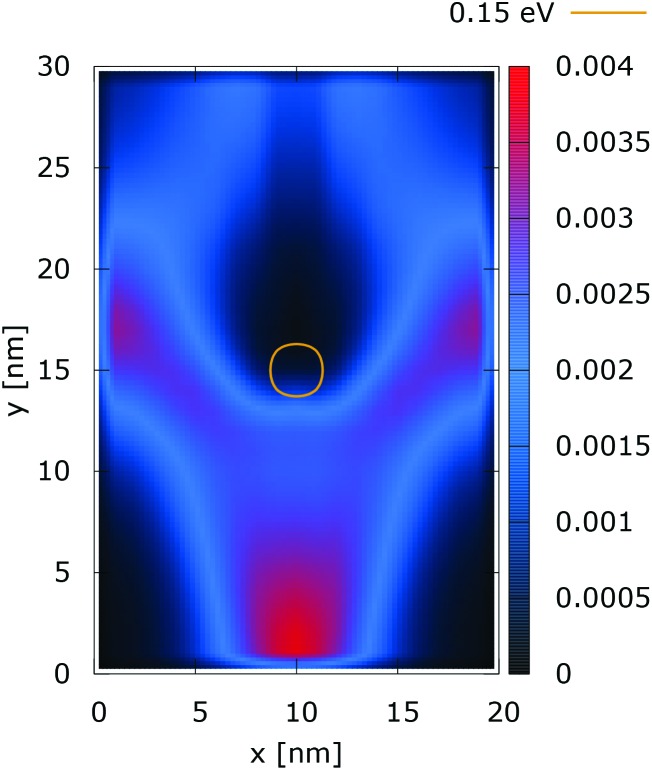
Quantum electron density [a.u.] with lateral reflecting boundaries. The dopant potential is 0.350 eV.

**Fig. 17:**
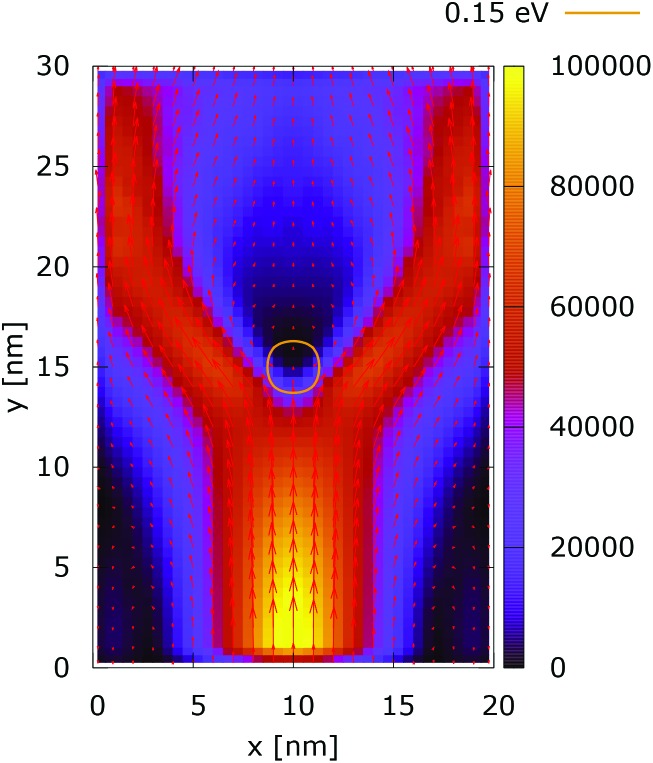
Classical current density [a.u.] with lateral reflecting boundaries. The dopant potential is 0.350 eV.

**Fig. 18:**
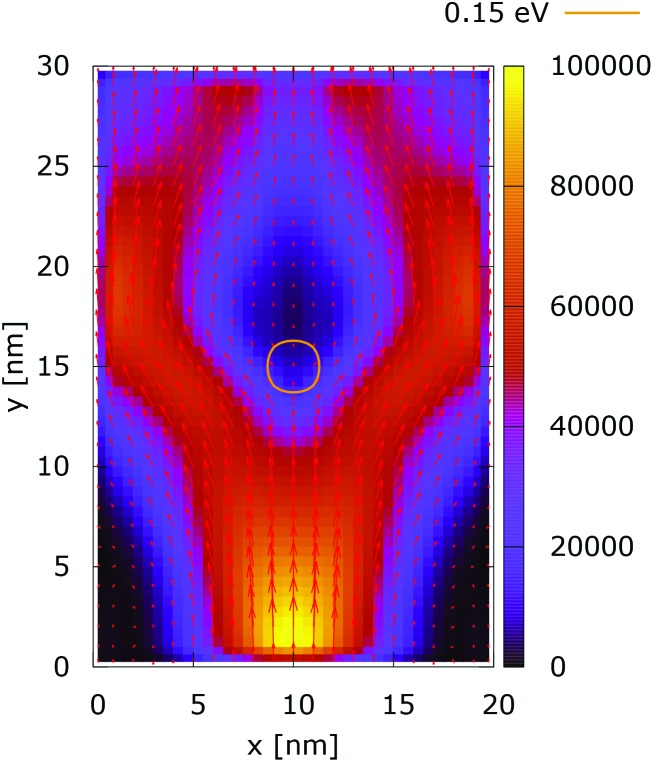
Quantum current density [a.u.] with lateral reflecting boundaries. The dopant potential is 0.350 eV.

These considerations are supported by the simulation results presented for the case of a 0.35 eV potential. The electron density distributions in Fig. 15 and 16 show that the lateral reflection happens further below the center, as a result of the stronger repulsion by the dopant. As a consequence of the non-local action of the potential in the quantum case, the reflection of the electrons from the lateral boundaries is observed much closer to the injecting boundary than in the classical counterpart. So, in the quantum case the electron density is much more concentrated around the dopant than both in classic and in the quantum 0.175 eV potential counterpart, shown in Fig. 12. This effect is better demonstrated by analyzing the corresponding current densities, shown in Fig. 17 and 18. In the quantum case the current density path is much more closed around the dopant than in the corresponding classical case. Moreover, comparing the current densities obtained with the 0.35 eV peak potential with those obtained with the 0.175 eV peak potential, Fig. 13 and 14, we can notice how the increase of the dopant potential changes the current density path more efficiently than in the corresponding classical cases.

The higher potential energy of the dopant generates also a low density current region in front of the dopant, as shown in Fig. 17, which corresponds to a low electron density region in Fig. 15.

We see that the effects due to the joint action of non-locality, tunneling, and repulsion are similar to the 0.175 eV case; however, more pronounced due the larger potential energy of the dopant.

These results motivate to investigate the effect of the interplay between these processes on the total current, discussed in the next section.

### Current analysis

The total current along *y*, obtained after integration of the two-dimensional current density *j*(*x*, *y*) with respect to the *x* coordinate10
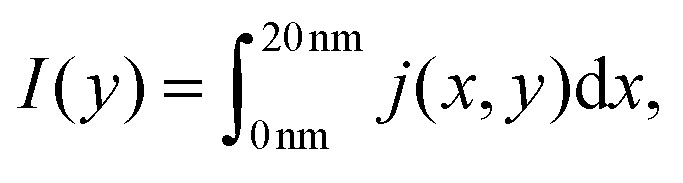
provides important information about the consistency of the simulations. In steady-state the density *I*(*y*) must be a constant as it follows from current-continuity considerations. Otherwise either the system is not in a steady-state, or the numerical approach is not reliable. We consider three cases

• constant potential;

• repulsive dopant with a peak potential of 0.175 eV;

• repulsive dopant with a peak potential of 0.35 eV.

**Fig. 19:**
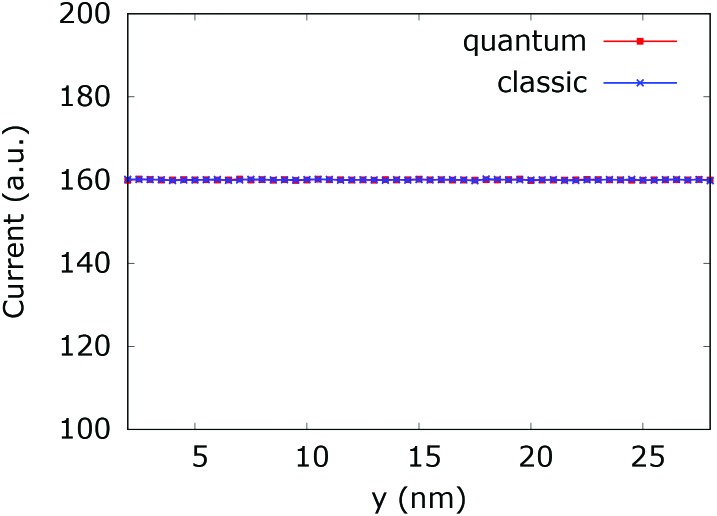
Comparison of the classical and quantum current in the quantum wire averaged between 250 fs and 400 fs. At constant potential the values coincide and are constant throughout the *y* axis.

Furthermore, under steady-state conditions, a time averaging of the calculated values increases the precision and the numerical stability of the simulations. We first check the consistency of our numerical approach. Fig. 19 shows the current inside the quantum wire without the repulsive dopant. In this case the classical current must coincide with the quantum counterpart. Indeed a constant potential means that there is no force acting on the classical electrons and no particle generation in the quantum case. As we already discussed, quantum and classical evolution coincide for up to quadratic potentials. A time averaging ensures a high precision of the stochastic results.

**Fig. 20:**
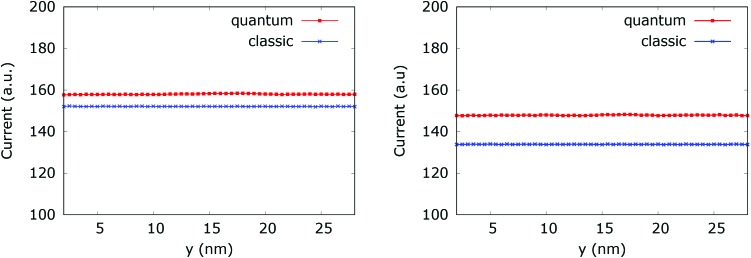
Comparison between the quantum and classical current in the quantum wire averaged between 250 fs and 400 fs with 0.175 eV repulsive dopant (left) and 0.35 eV (right).

Fig. 20 (left) shows the current for a 0.175 eV potential. In this case both classic and quantum currents decrease with respect to the case without the dopant. In particular we observe a 4.95% decrease of the classical current as compared to the current in the constant potential case. Instead in the quantum case the current decreases only by 1.26%. This means that the quantum current increase is about 3.9% higher than in the corresponding classical case. Fig. 20 (right) shows the current for a 0.35 eV potential. The higher dopant potential gives rise to a further decrease of both the classical and quantum current densities. But there is also a further increase of the difference between the two counterparts. The classical current decreases by 16.35%, while the quantum current decreases by 7.6% with respect to the constant potential case, giving rise to a 10.47% difference in favor of the interplay between tunneling, non-locality carried by the higher order potential derivatives in (4), and the boundary reflection.

Finally, we note that the fringes, which can be observed in the V-shaped region behind the impurity (see in particular Fig. 5, 9 and 16) are a manifestation of quantum interference. The latter is much more strongly pronounced in the case of two dopants.^9^ In particular, the specular reflection from the boundaries maintains the quantum coherence as shown by an analysis of the purity of the evolution.^52^

## 5. Conclusions

This research focuses on quantum processes in quantum wires hosting a dopant in the electron transport path, which is an important contribution to further the understanding of novel device operation concepts for entangletronics. The presented analysis of the surfacing coherence effects in the evolution of electrons around a single repulsive dopant uses as a reference frame the corresponding classical picture. Very convenient for this study is the Wigner particle approach, which treats classical and quantum evolution processes on an equal footing. The interplay of the three elemental phenomena quantum non-locality, tunneling, and boundary reflection can be quite complicated. Any of these phenomena may lead to a small modification of the physical averages; however, their joint action can have an impact on the latter. A 10 percent increase of the mean current is considered a good improvement from an engineering point of view.

The above considerations in particular illustrate why it is so difficult to explore the world of quantum electron transport: the interplay of quantum amplitudes does not allow for a separate treatment of the involved phenomena as is the case of classical accumulation of probabilities. A global treatment is needed and the results can be different for close physical setups as illustrated by the considered density and current behavior. The situation becomes even more complicated if we include quantum penetration and surface roughness in the physical picture.

## Conflicts of interest

There are no conflicts to declare.
